# Life-threatening arrhythmias from dynamic LVOT obstruction by a large left ventricular fibroma in an adolescent: complete resolution after combined transaortic-transatrial resection—a case report

**DOI:** 10.3389/fcvm.2026.1772382

**Published:** 2026-03-17

**Authors:** Ming-Liang Dong, Qingbao Li, Jie Zi, Hongxin Li

**Affiliations:** 1 Department of Cardiovascular Surgery, Shandong Provincial Hospital, Shandong University, Jinan, China; 2 Department of Cardiovascular Surgery, Shandong Provincial Hospital Affiliated to Shandong First Medical University, Jinan, China; 3 Department of Cardiovascular Surgery, Shandong Provincial Qianfoshan Hospital, Shandong University, Jinan, China

**Keywords:** case report, left ventricular fibroma, surgical resection, syncope, ventricular fibrillation

## Abstract

Cardiac fibromas are benign tumors that infrequently cause hemodynamic compromise or arrhythmias, particularly in adolescents. We describe a 15-year-old male who presented with nonspecific symptoms evolving into recurrent dizziness and syncope due to a large left ventricular (LV) fibroma obstructing the LV outflow tract (LVOT). Complicated by ventricular fibrillation arrest, the case underscores the potential for life-threatening events. Surgical excision via a combined transaortic and transatrial approach resulted in complete resolution, with no residual mass prior to discharge. This report highlights the diagnostic challenges, surgical strategy, and favorable prognosis, emphasizing early intervention in symptomatic pediatric cardiac tumors.

## Introduction

Primary cardiac tumors are exceedingly rare, with an incidence of 0.0017%–0.28% in autopsy series (1), and fibromas representing the second most common benign type after rhabdomyomas in pediatric populations (2). Typically arising in the LV free wall or septum, fibromas consist of fibrous tissue and can lead to arrhythmias, heart failure, or outflow tract obstruction, manifesting as syncope or sudden cardiac events (1). While often asymptomatic and discovered incidentally, symptomatic cases, especially those with LVOT involvement, warrant surgical intervention to avert complications like ventricular tachycardia or fibrillation (3). Literature reviews indicate that syncope occurs in approximately 15% of cases, frequently linked to arrhythmias rather than pure obstruction (3). Obstructive presentations mimicking hypertrophic cardiomyopathy are documented but rare in adolescents (2, 4). Beyond mechanical obstruction, emerging evidence suggests that the dense collagenous matrix in fibromas may create zones of slowed conduction and unidirectional block, fostering re-entrant arrhythmias even in the absence of overt hemodynamic compromise (5). This intrinsic arrhythmogenic potential underscores the need for vigilant monitoring in adolescents, where fibromas can pose a heightened risk of sudden cardiac events despite their benign histology (6). This case illustrates a unique intersection of dynamic LVOT obstruction, recurrent syncope, and arrhythmic arrest in a teenager, managed successfully through multidisciplinary care.

## Case presentation

This case is reported in accordance with the CARE guidelines (7). The completed checklist is available as Supplementary Material S1. A 15-year-old previously healthy male presented to the emergency department on November 5, 2025, with a 1-month history of nasal congestion, cough, and intermittent headaches following exercise and sweating. Symptoms progressed to chest tightness and pain 1 week prior, exacerbated by running, with associated dizziness and transient blackout. Upon admission, he demonstrated unsteadiness after exertion but retained normal appetite, sleep, and bowel habits, with stable weight. He reported no fever, nausea, vomiting, or prior cardiac issues. The patient had no significant family history of congenital heart disease, sudden cardiac death, or known genetic syndromes. There was no parental consanguinity.

Physical examination revealed no precordial bulge, normal apical impulse, and unexpanded cardiac dullness. Heart rate was 75 beats per minute, rhythm regular, with a grade 3/6 systolic ejection murmur in the aortic valve area accompanied by a thrill. Laboratory evaluations showed hypokalemia (3.10 mmol/L), elevated high-sensitivity troponin T (42.70 pg/mL), hypoalbuminemia (total protein 61.0 g/L, albumin 41.1 g/L), and mild thrombocytopenia (127 × 10^9^/L).

**Figure 1:**
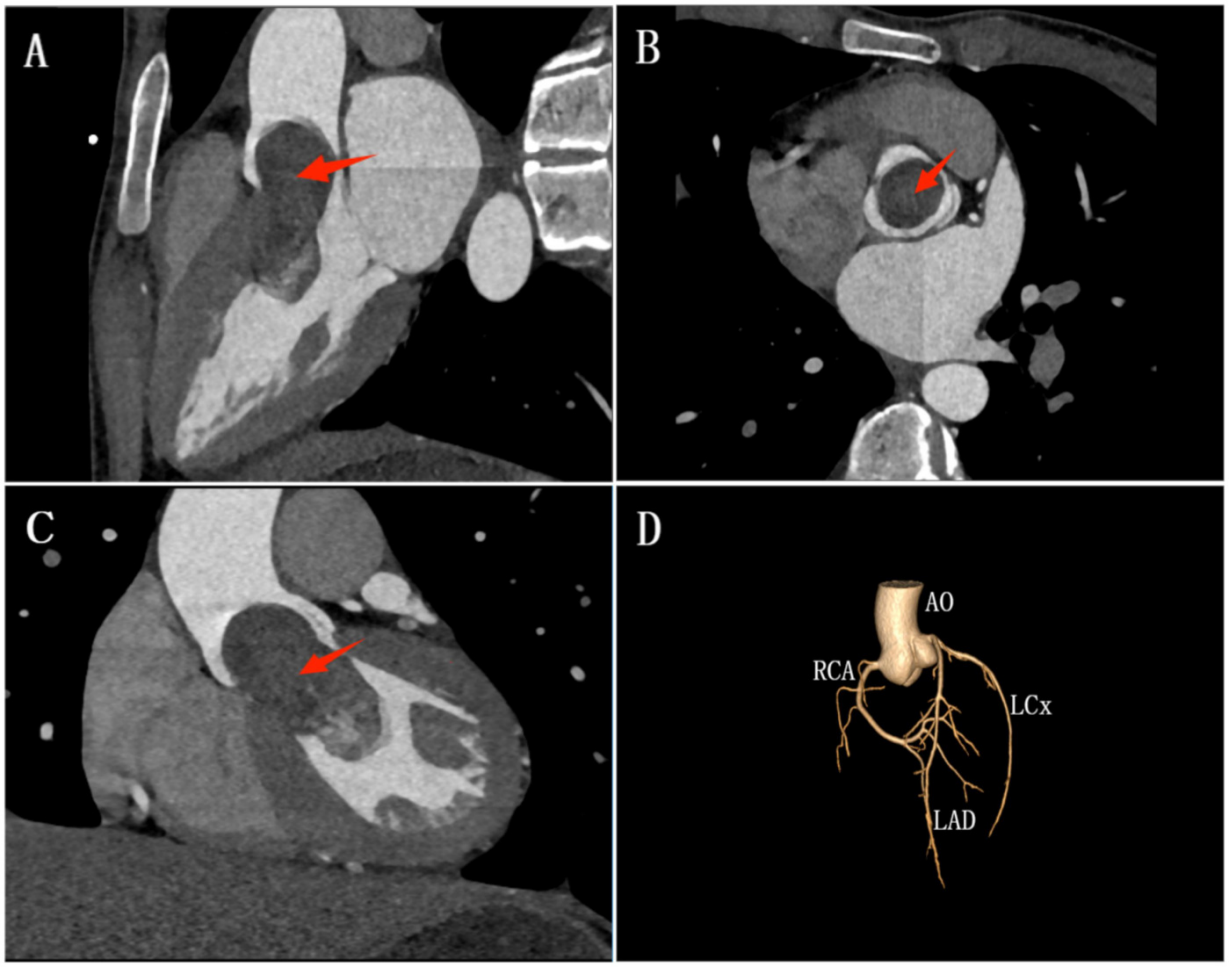
CTA imaging of the left ventricular fibroma causing dynamic LVOT obstruction. Preoperative CTA offered detailed visualization of the left ventricular fibroma across sagittal, horizontal, and coronal planes (red arrow), revealing its dimensions measuring approximately 5.22 cm × 2.48 cm × 2.34 cm **(A–C)**. Subsequent 3D coronary reconstruction clearly delineated all branches, revealing no evidence of vascular compression or invasion by the fibroma and no signs of blood flow obstruction **(D)**.

Transthoracic echocardiography revealed mild LV enlargement with a heterogeneous mass attached via a pedicle to the mid-interventricular septum. During systole, the mass prolapsed into the LVOT, partially crossing the aortic valve, resulting in obstruction and segmental hypokinesia of the mid-septum. Color Doppler demonstrated turbulent flow with a peak gradient of approximately 99 mmHg. Coronary computed tomography angiography (CTA) excluded stenoses in major vessels but confirmed a low-density LV mass extending into the ascending aorta during systole, with no valvular calcification or pericardial effusion. No significant diagnostic challenges were encountered; the diagnosis of left ventricular fibroma with dynamic LVOT obstruction was clearly established by multimodality imaging (Figure 1).

On November 10, 2025, at 01:30, the patient developed ventricular fibrillation, prompting immediate resuscitation. Cardiopulmonary resuscitation and defibrillation (150 J) restored sinus rhythm by 01:50, with post-arrest blood pressure 90/60 mmHg. Arterial blood gas analysis indicated hyperlactatemia (5.40 mmol/L), hypocapnia (PCO_2_ 27.00 mmHg), and metabolic acidosis (base excess −6.40 mmol/L, total CO_2_ 17.50 mmol/L).

**Figure 2:**
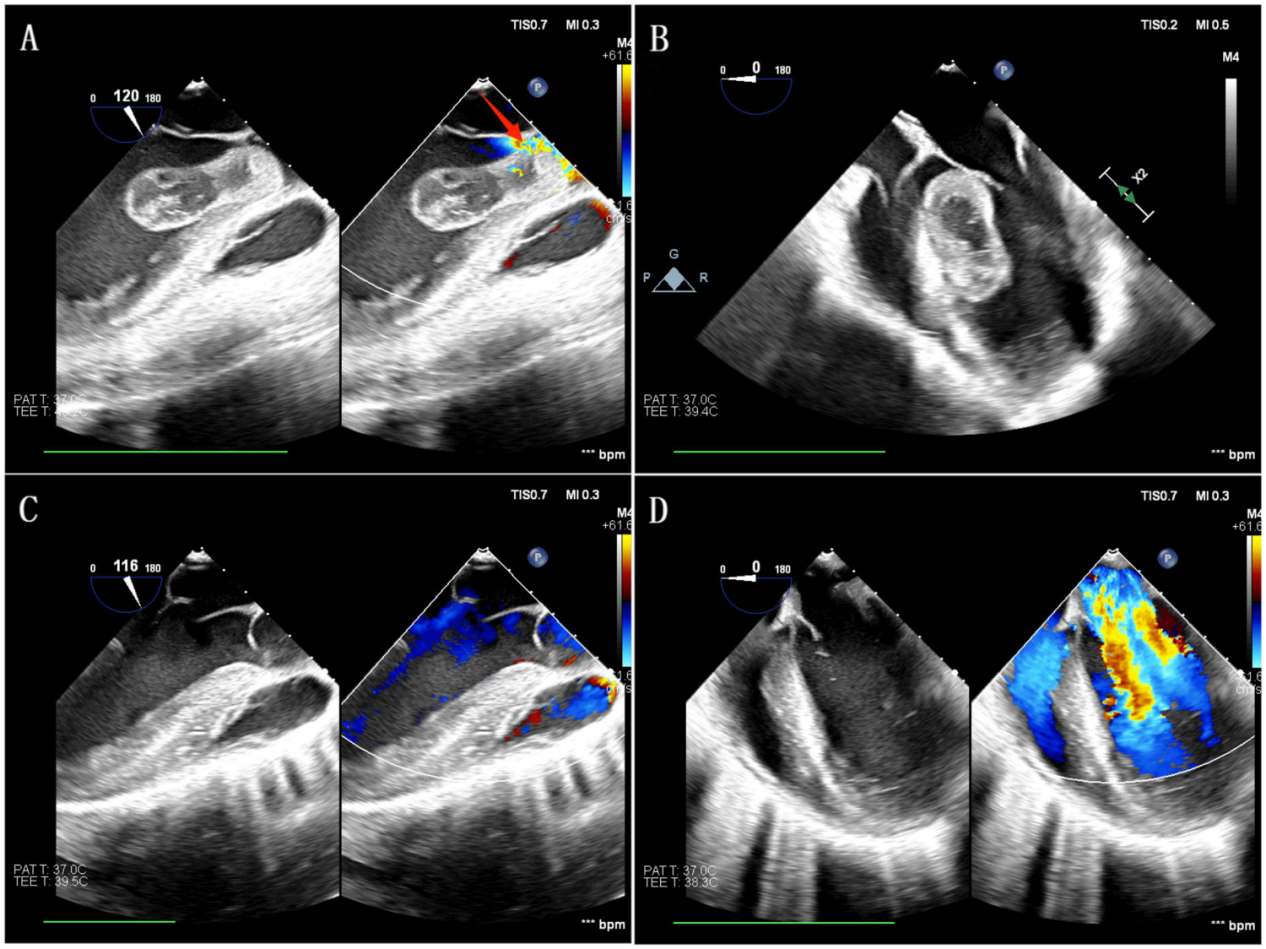
TEE findings and confirmation of complete resection. Intraoperative TEE revealed a tumor projecting prominently into the left ventricular outflow tract during systole (long-axis view), partially crossing the aortic valve and causing severe outflow tract obstruction (as indicated by the red arrow). Continuous Doppler measurements demonstrated a peak left ventricular outflow tract flow velocity of 4.94 m/s and a mean transvalvular pressure gradient of 99 mmHg, with prominent high-velocity turbulent mixed signals **(A)** The tumor was attached to the mid-ventricular septum via a pedicle. During diastole (four-chamber view), the mass retreated into the left ventricle and significantly obstructed the opening of the mitral valve's anterior leaflet **(B)** Postoperative TEE demonstrated successful resolution of left ventricular outflow tract obstruction, with complete disappearance of subaortic outflow tract color turbulence signals. The maximum flow velocity in the ventricular outflow tract decreased to 0.95 m/s, and the mean transvalvular pressure difference was 4 mmHg **(C)** The ventricular septum remained continuous and intact, with homogeneous surface echogenicity. The anterior leaflet of the mitral valve could be fully opened without restriction **(D)**.

In an emergency, surgical exploration under cardiopulmonary bypass was performed, involving an aortotomy that revealed a massive tumor obscuring critical boundaries. Extension through right atriotomy and atrial septal incision exposed a 5 cm × 3 cm mass with a 1 cm broad pedicle near the posterior papillary muscle on the ventricular septum. Complete excision was achieved via combined aortic and mitral valve access, followed by electrocautery of the attachment site and LV irrigation with iced saline to clear debris. No residual tumor was noted in the mitral valve, left atrial appendage, or pulmonary veins. The atrial septum and aorta were closed with 4-0 Prolene sutures (Table 1). Postoperative transesophageal echocardiography (TEE) during surgery confirmed mass removal and unobstructed flow (Figure 2).

**Table 1:** Timeline of key events.

Date	Event	Key findings/Interventions
10.28.2025	Onset of nasal congestion, cough, headache post-exercise	No treatment initially
11.3 2025	Progression to chest pain, dizziness, blackout after running	Aztreonam at local hospital, ineffective
11 5, 2025	Admission to hospital	Hypokalemia, elevated troponin; murmur noted
11 6, 2025	Laboratory follow-up	Decreased troponin, hypoalbuminemia, thrombocytopenia
11 7, 2025	Preoperative CTA	LV mass with LVOT obstruction confirmed
11 10, 2025	Ventricular fibrillation arrest	CPR, defibrillation; supportive care
11 10, 2025	Surgical resection	Complete tumor removal via combined approach
11 14, 2025	Postoperative echocardiography	No residual mass, normal function

The perioperative course was uneventful, without any surgical complications, such as bleeding, infection, low cardiac output, or new—onset arrhythmias. The patient tolerated the procedure well, having no issues regarding adherence to postoperative care or intolerance to the surgical intervention. Transthoracic echocardiography performed on postoperative day 4 demonstrated complete resection with no fibroma, resolution of LVOT obstruction, and preserved left ventricular function. The patient was discharged on postoperative day 8 in stable condition.

**Figure 3:**
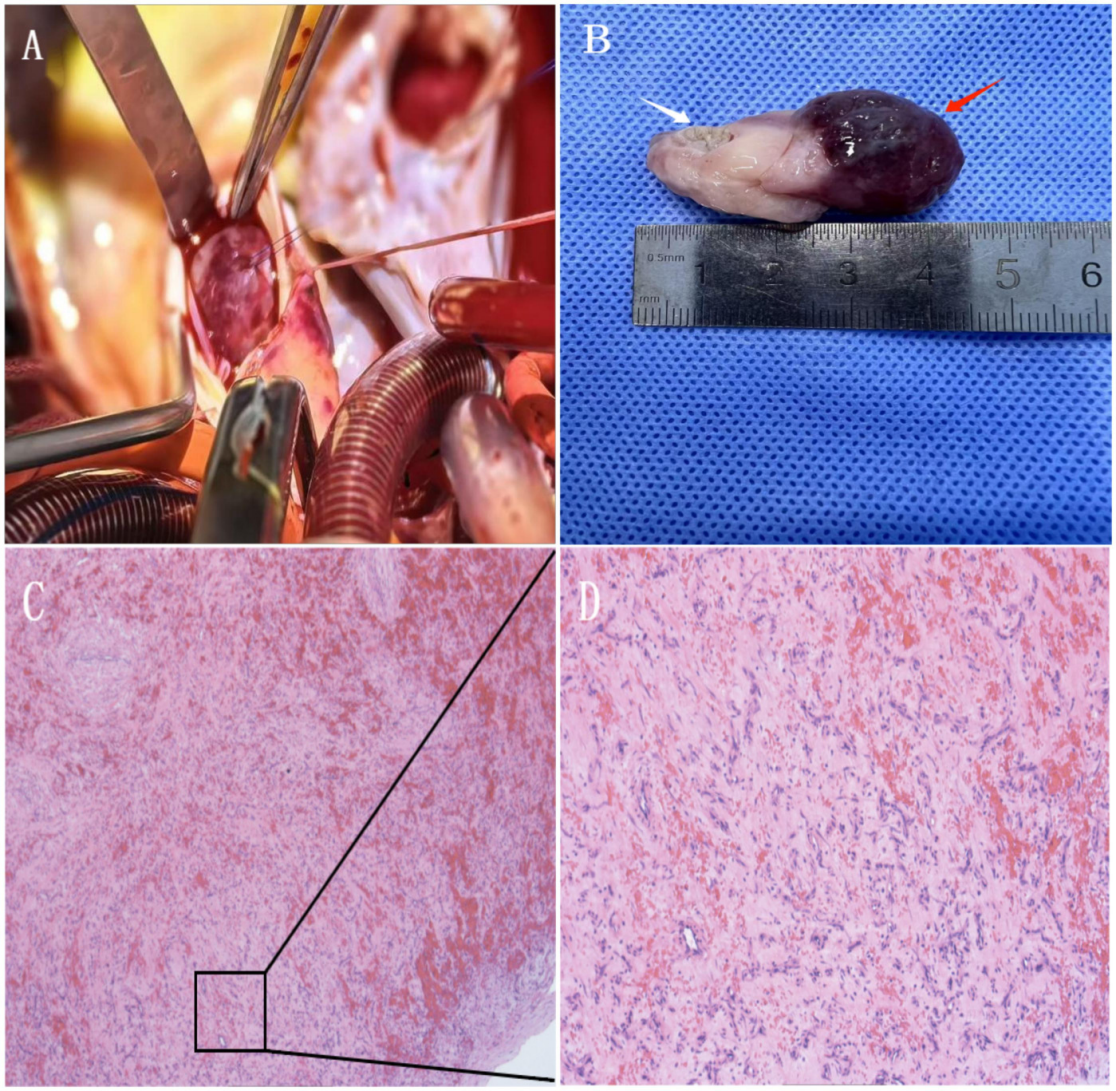
Histopathological confirmation of cardiac fibroma. Intraoperative view after aortotomy showing the tumor extending into the LVOT **(A)**. Resected specimen demonstrating a hematoma at the free end (red arrow) and the distal pedicle (white arrow) **(B)**. Histological sections (H&E staining) revealing dense collagenous tissue with cystic degeneration, vascular proliferation, focal hemorrhage, and thrombosis **(C,D)**.

The resected specimen measured approximately 5 cm × 3 cm with a 1-cm broad pedicle attached to the mid-ventricular septum near the posterior papillary muscle. Gross examination revealed a firm, whitish mass with a hematoma at the free end (Figures 3A,B). Histopathological examination confirmed the diagnosis of cardiac fibroma, showing dense collagenous fibrous tissue with cystic degeneration, vascular hyperplasia, focal hemorrhage, thrombosis, and organization (Figures 3C,D).

## Discussion

The present case vividly illustrates a life-threatening presentation of a cardiac fibroma in an adolescent, where dynamic LVOT obstruction and recurrent syncope culminated in ventricular fibrillation—a rare but catastrophic complication (8). We hypothesize that the fatal arrhythmia in our patient resulted from the convergence of a chronic arrhythmogenic substrate and an acute hemodynamic insult. Crucially, the intraoperative discovery of a hematoma at the free end of the resected tumor suggests a pivotal event: acute intratumoral hemorrhage or vascular rupture. This likely caused abrupt mass expansion, leading to critical dynamic LVOT obstruction, a sharp drop in cardiac output, and ultimately, serving as the final trigger for ventricular fibrillation. This acute-on-chronic pathophysiology is supported by the histopathological findings of vascular hyperplasia, focal hemorrhage, and thrombosis within the fibroma (9), underscoring that even histologically benign tumors can precipitate sudden cardiac death through volatile structural changes. Strengths of this case report include the detailed correlation of multimodal imaging (echocardiography and CTA) with intraoperative and histopathological findings, the successful application of a combined transaortic-transatrial surgical approach for complete resection of a broad-based tumor, and documentation of both short-term clinical resolution and imaging confirmation postoperatively. These elements provide valuable insights into the management of rare, life-threatening presentations of cardiac fibromas in adolescents.

These histopathological features not only elucidated the acute triggering event but also definitively confirmed the diagnosis of fibroma, distinguishing it from other primary cardiac tumors. Differential diagnoses included myxoma or rhabdomyoma, but imaging and pathology favored fibroma (10). Key distinguishing features supported the diagnosis of fibroma over myxoma or rhabdomyoma. Unlike myxomas, which are typically atrial-based with a pedicle and heterogeneous appearance due to hemorrhage or necrosis, fibromas are intramural, predominantly ventricular, and often demonstrate calcification on CT (11). Rhabdomyomas, more common in infancy and often multiple with associations to tuberous sclerosis, tend to lack calcification and may spontaneously regress, contrasting with the solitary, non-regressing nature of fibromas (12).

With the diagnosis of fibroma firmly established, the tumor's broader chronic arrhythmogenic implications warrant further consideration. Cardiac fibromas possess an intrinsic, chronic arrhythmogenic potential that mandates their recognition not merely as space-occupying lesions but as active arrhythmogenic foci. The dense collagenous matrix and the associated fibrotic remodeling create a permanent pro-arrhythmic substrate by disrupting uniform conduction and promoting anisotropic propagation, where electrical impulses are delayed or blocked transversely, thus fostering re-entrant circuits such as ventricular tachycardia (13). Cardiac fibrosis in fibromas typically reduces myocardial conduction velocity by approximately 20%–50%, as observed in fibrotic models, exacerbating conduction heterogeneity and reentry (14). Furthermore, chronic microvascular ischemia and altered cellular electrophysiology in the peritumoral zone may lower the threshold for triggered activity (5). This perspective is crucial for managing asymptomatic or mildly symptomatic patients, as it warrants consideration of proactive surgical resection in selected cases. The decision to intervene should be individualized, incorporating factors such as tumor location, size, and documented arrhythmias. Long-term arrhythmia surveillance is equally important, for which implantable loop recorders offer a valuable tool, as illustrated in specific cases for continuous monitoring (15). Building on this mechanistic understanding, surgical strategies must address both the mechanical obstructive effects and the underlying electrical substrates to achieve definitive resolution.

For symptomatic fibromas, surgical resection is curative. The combined transaortic-transatrial approach employed in this case provided optimal exposure for the safe and complete excision of the broad-based, pedunculated tumor, minimizing risks such as incomplete resection or embolization. The literature consistently reports high rates of symptom resolution and an exceedingly low recurrence rate following complete resection. Contemporary studies with mid- to long-term follow-up demonstrate that surgical resection can be performed with excellent survival and low risk of adverse events. A large single-center series reported no mortality and a 15-year risk of clinical ventricular tachycardia/fibrillation of only 2.4% after resection in children (16). Another study focusing on pediatric cardiac tumors reported an 8-year survival rate of 90.4% following surgery, with resolution of arrhythmias in the majority of patients (17). Recent analyses further indicate that post-resection ventricular arrhythmias persist in up to 10% of adult cases compared to 2%–5% in pediatric cohorts, highlighting age-related differences in arrhythmic substrates (18).

In summary, this case reinforces a pivotal clinical concept: cardiac fibromas, particularly in adolescent patients, should be recognized as dual-threat lesions possessing both mechanical obstructive potential and intrinsic arrhythmogenicity. Consequently, clinical management should not rely solely on symptom severity. We recommend a multidisciplinary team risk assessment for all adolescent patients. Proactive consideration of surgical resection, especially in the presence of risk factors such as arrhythmias or significant obstruction, coupled with vigilant long-term post-operative surveillance (for which implantable loop recorders offer a valuable tool), is paramount to mitigate the risk of sudden cardiac death.

## Limitations

Due to the urgent clinical presentation and the imminent risk of hemodynamic collapse from dynamic LVOT obstruction, the preoperative window was extremely short. The primary focus was rapid diagnosis and stabilization, precluding the performance of 24-hour ambulatory electrocardiogram (ECG) monitoring or invasive electrophysiological studies. Consequently, no sustained arrhythmic episodes (such as non-sustained ventricular tachycardia or frequent ventricular ectopy) were documented prior to the ventricular fibrillation arrest on November 10, 2025. The primary limitation of this report remains the relatively short follow-up period. Therefore, we strongly advocate for long-term arrhythmia surveillance, given the well-documented association of cardiac fibromas with ventricular ectopy even after complete resection.

## Patient perspective

The patient's legal guardian reported that the recurrent episodes of dizziness and syncope had caused considerable family anxiety and significantly restricted the adolescent's daily activities and school participation. Following surgery, the guardian noted complete resolution of symptoms and expressed profound gratitude for the rapid diagnosis and expert intervention, stating that the family now feels confident allowing the patient to resume normal physical activities without fear of sudden collapse. This outcome has markedly improved the patient's and family's quality of life.

## Conclusion

This case demonstrates the critical role of imaging in diagnosing obstructive LV fibromas and the efficacy of surgical intervention in preventing fatal arrhythmias. Prompt recognition and excision can yield excellent prognosis in adolescents.

## Data Availability

The datasets presented in this article are not readily available because of ethical and privacy restrictions. Requests to access the datasets should be directed to the corresponding authors.
